# Comprehensive Assessment of Neuropathy and Metabolic Parameters in Type 1 Diabetic Patients with or Without Using Continuous Glucose Sensors

**DOI:** 10.3390/ijms26052062

**Published:** 2025-02-26

**Authors:** Barbara Bordács, Ákos Várkonyi, Zsuzsanna Valkusz, Szabolcs Nyiraty, Anikó Pósa, Adrienn Menyhárt, Csaba Lengyel, Péter Kempler, Krisztina Kupai, Tamás Várkonyi

**Affiliations:** 1 Department of Internal Medicine, Albert Szent-Györgyi Medical School, University of Szeged, 6725 Szeged, Hungary; bordacs.barbara.aniko@med.u-szeged.hu (B.B.); valkusz.zsuzsanna@med.u-szeged.hu (Z.V.); nyiraty.szabolcs@med.u-szeged.hu (S.N.); lengyel.csaba@med.u-szeged.hu (C.L.); varkonyi.tamas@med.u-szeged.hu (T.V.); 2 Faculty of Dentistry, University of Szeged, 6720 Szeged, Hungary; szenzor.varkonyiakos@gmail.com (Á.V.); aniko.posa@icloud.com (A.P.); 3 Department of Oncology and Internal Medicine, Faculty of Medicine, Semmelweis University, 1083 Budapest, Hungary; menyhartadri@gmail.com (A.M.); kempler.peter@semmelweis.hu (P.K.)

**Keywords:** diabetes mellitus, continuous glucose monitoring, CGM, neuropathy, triglyceride, serum lipid, cholesterol

## Abstract

The present study was conducted in type 1 diabetic (T1DM) patients to evaluate the metabolic and glycemic control as well as the manifestations of neuropathy. The impact of continuous glucose monitoring (CGM) on the measured parameters was also analyzed. A total of 61 T1DM patients (age: 42.5 ± 1.8 years, DM duration: 22.8 ± 1.6 years, mean ± SE) participated in the study. In total, 24 patients had CGM sensors and 37 did not. Cardiovascular autonomic neuropathy was assessed using cardiovascular reflex tests. Peripheral sensory function was evaluated by a Neurometer and calibrated tuning fork on the upper and lower limbs. Metabolic status was characterized by the determination of triglycerides, high-density lipoprotein (HDL), low-density lipoprotein (LDL), total cholesterol, and glycated haemoglobin (HbA_1c_). A positive correlation was found between HbA_1c_ and triglyceride levels (r = 0.28, *p* < 0.05). CGM users and non-users differed in triglyceride (0.9 ± 0.1 vs. 1.24 ± 0.12 mmol/L, *p* < 0.05), HDL cholesterol (1.7 ± 0.1 vs. 1.4 ± 0.1 *p* < 0.05 mmol/L), and HbA_1c_ (7.5 ± 0.2 vs. 8.3 ± 0.3%, *p* < 0.05) levels as well. Significant differences were found for the Valsalva ratio, Neurometer, and calibrated tuning fork results between CGM users and non-users. This study found a significant correlation between HbA_1c_ and triglyceride levels in T1DM. CGM use resulted in improved metabolic parameters and less autonomic and sensory nerve damage. As a novel finding, CGM is presumed to prevent both micro-, and macrovascular complications and, by this way, potentially reducing mortality rates.

## 1. Introduction

**Figure 1:**
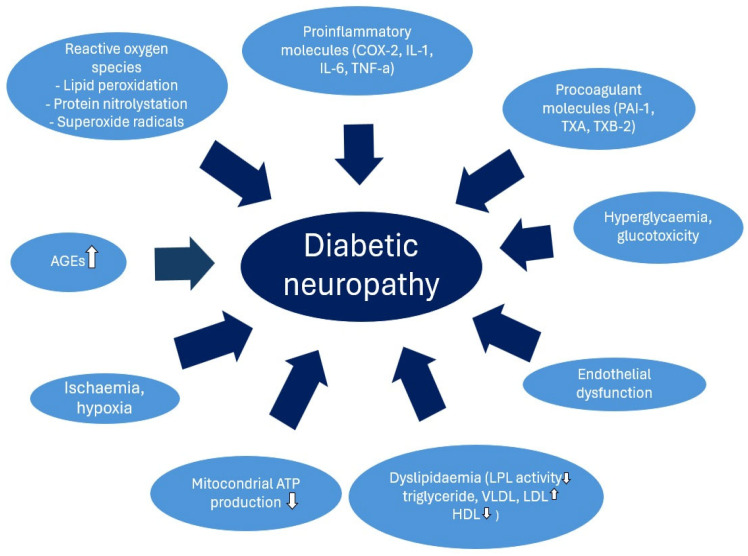
Pathomechanism of diabetic neuropathy.

Diabetes mellitus is a complex metabolic disease that primarily affects carbohydrate metabolism but its impact on lipid and protein metabolic processes cannot be neglected. Compared to the healthy population, patients with diabetes have a higher risk of cardiovascular complications and mortality, particularly due to the elevated levels of detrimental metabolic parameters that contribute to atherosclerosis [1]. Cardiovascular autonomic neuropathy (CAN) and distal symmetric polyneuropathy (DSPN) are frequent complications of diabetes, mainly in individuals with prolonged periods of elevated glucose levels. In addition to hyperglycemia, several associated factors, including inflammation, oxidative stress, endothelial dysfunction, advanced glycated end products (AGEs), hypoxia, and ischemia, are implicated in their pathogenesis. Among these, the role of dyslipidemia is particularly significant (Figure 1) [2,3].

Diabetic neuropathy severely impacts the quality of life of patients by causing lower extremity pain, leg ulceration, an increased need for amputations, and higher mortality rates in both type 1 (T1DM) and type 2 diabetic (T2DM) patients. Impaired sympathetic and parasympathetic autonomic function due to neuropathy contributes significantly to the elevated incidence of cardiovascular events and increased mortality in diabetic individuals. The risk of death is much higher in cases of hyperlipidemia as well, especially in patients with T2DM. A significant correlation was established between CAN and age, diabetes duration, higher glycated hemoglobin (HbA_1c_), fasting triglyceride (TG), and lower high-density lipoprotein (HDL) levels in T1DM patients [4]. HbA_1c_ is associated with dyslipidemia, including elevated low-density lipoprotein (LDL) and TG [5], and is also an important parameter in predicting coronary artery disease in T2DM subjects [6]. Patients with T1DM are also at higher risk of developing any diabetes-related complications and all-cause mortality. Inadequate glycaemic control and glucose variability are associated with higher cholesterol, LDL, and TG levels and lower HDL levels [7].

A recent meta-analysis found that continuous glucose monitoring (CGM) metrics might determine not only the microvascular but also the macrovascular complications in T1DM [8]. The use of HbA_1c_ alone is not sufficient to determine the effectiveness of therapy as it is a measure of average blood glucose levels and does not provide information on episodes of hyperglycemia or hypoglycemia. Capillary blood glucose measurements are usually taken at different random time points and, thus, cannot be relied upon for accurate data. The use of CGM has significantly increased in recent years and decades. Several studies have shown the benefits of CGM in diabetic patients by providing real-time blood glucose values, thus enhancing the management of glycaemic therapy [9,10]. It is widely accepted, that CGM plays a key role in improving carbohydrate metabolism and achieving optimal HbA_1c_. However, its impact on micro- and macrovascular complications of diabetes and lipid metabolism is still poorly understood. To explore the details of the relationship between the components of the complex metabolic control and diabetic neuropathy in T1DM, we organized a trial in which we evaluated the diabetic autonomic and peripheral sensory functions as well as the glycemic and lipid parameters. We focused on the role of the application of CGM as a new potential tool in the improvement of metabolic control and preservation of neuronal function.

## 2. Results

### Synthesis of Results

A total of 61 patients (age: 42.5 ± 1.8 years, DM duration: 22.8 ± 1.6 years, BMI: 25.3 ± 0.9, HbA_1c_: 8.1 ± 0.2%; mean ± SE) were studied (Table 1). In total, 24 patients used CGM sensors (age: 35.7 ± 2.5, DM duration: 20.0 ± 1.8, BMI: 24.5 ± 0.8, HbA_1c_: 7.5 ± 0.8%) and 37 did not (age: 45.9 ± 1.8, DM duration: 25.0 ± 1.9, BMI: 26.5 ± 0.8, HbA_1c_: 8.3 ± 0.3%, Table 2). A total of 19 of the CGM user patients had been wearing sensors for more than 4 years while 5 of them used sensors for between 6 months and 4 years.

**Figure 2:**
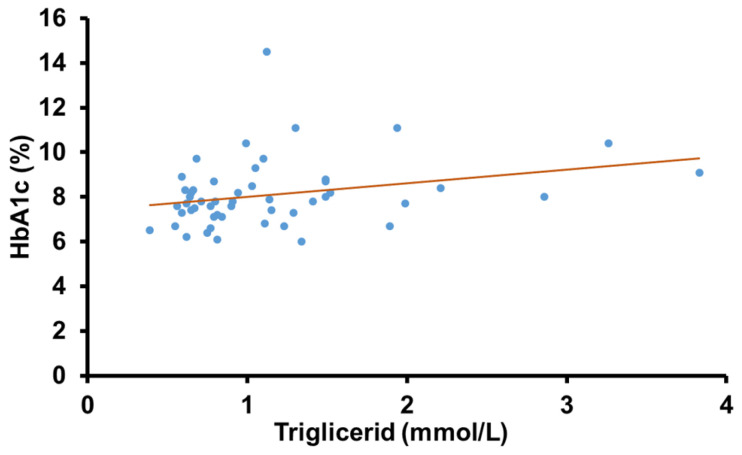
Correlation between HbA_1c_ and triglyceride levels in T1DM patients.

A significant positive correlation was found between HbA_1c_ and TG levels in the overall group of T1DM patients (r = 0.28, *p* = 0.045) (Figure 2). The mean lipid parameters were within the normal range. However, neither the HbA_1c_ nor the lipid parameters showed a significant correlation with the cardiovascular autonomic or peripheral sensory function (*p* > 0.05 in all cases).

For further analysis, the diabetic group was divided into two subgroups based on CGM usage. The evaluation of the cardiovascular autonomic reflex tests revealed a significant difference in VR values between CGM users and non-users (1.38 ± 0.06 vs. 1.27 ± 0.04, *p* = 0.045, Table 3). No significant differences were observed in the results of the remaining three tests (Table 3) although results were more physiological in the CGM group.

Using the Neurometer, we found a significant difference in large sensory nerve fiber function between CGM users and non-users, specifically in the perception thresholds at the median nerve during 2000 Hz stimulation (224.4 ± 21.2 vs. 290.6 ± 17.7, *p* = 0.01). No significant differences were observed in the results of further tests on the median and peroneal nerves between the CGM users and non-users (Table 4).

Calibrated tuning fork tests revealed significant differences in the vibration sense between CGM users and non-users at the right radius (7.4 ± 0.1 vs. 7.1 ± 0.1, *p* = 0.01) and the right hallux (7.2 ± 0.2 vs. 6.1 ± 0.3, *p* = 0.005, Table 5). However, five out of six CPT values were higher, indicating some degree of hypesthesia among non-CGM users.

Additionally, CGM users exhibited significantly different metabolic parameters, including lower TG (0.9 ± 0.1 vs. 1.2 ± 0.1 mmol/L, *p* = 0.034) and HbA_1c_ (7.5 ± 0.2% vs. 8.3 ± 0.3%, *p* = 0.04) levels, as well as higher HDL cholesterol levels (1.7 ± 0.1 vs. 1.5 ± 0.1 mmol/L, *p* = 0.02), compared to non-users (Table 6).

## 3. Discussion

Several studies have explored the relationship between glycemic control, diabetic complications, and lipid metabolism in patients with T2DM but much less evidence is available on T1DM [11]. Unlike previous research, our study aimed to investigate the potential relationships among the glycemic and lipid parameters as well as the autonomic and peripheral neuronal dysfunctions in T1DM patients. Recognizing that CGM offers an opportunity for improved glycemic control, we also focused on comparing these parameters between CGM users and non-users.

A clear positive correlation was found between HbA_1c_ and fasting TG levels among our participants. Previous research demonstrated that TG does not impair, in a clinically significant manner, the performance of HbA_1c_ measurement so the correlation is not due to a methodological problem [12]. It is published in the literature that elevated TG levels are associated with inadequate glycemic control in T2DM and it is explained mainly by the pathogenetic role of insulin resistance in these patients [13]. Hypertriglyceridemia observed in T1DM patients may be attributed to increased VLDL production secondary to relative insulin deficiency [14] and decreased lipoprotein lipase activity in patients inadequately treated with insulin [7]. Both hyperglycemia and hyperlipidemia can exacerbate oxidative stress, endothelial dysfunction, and inflammation, leading to a multiplicity of cardiovascular risks in T1DM patients. Given the significant correlation between HbA_1c_ and TG levels, either parameter may serve as a predictor of metabolic control in T1DM. Moreover, our findings highlight that elevated TG levels are not exclusively a sign of insulin resistance but are also associated with worse glycemic control in insulin-dependent patients.

As CGM usage means a promise of better glycemic control in both types of diabetes, we divided our T1DM patients into two groups based on CGM use. Our findings revealed that patients not using CGM exhibited significantly higher HbA_1c_ and fasting TG levels, whereas CGM users demonstrated higher HDL levels. The more optimal glycaemic control, as a result of utilizing CGM, seems to positively influence lipid profiles, possibly through preventing the extreme fluctuations of glucose, promoting a more stable glycemic condition by reducing hypoglycemic episodes and enabling timely management of hyperglycemia with additional insulin boluses. The reduced glucovariability potentially lowers the risk of vascular complications primarily through the reduction of oxidative stress [15].

**Figure 3:**
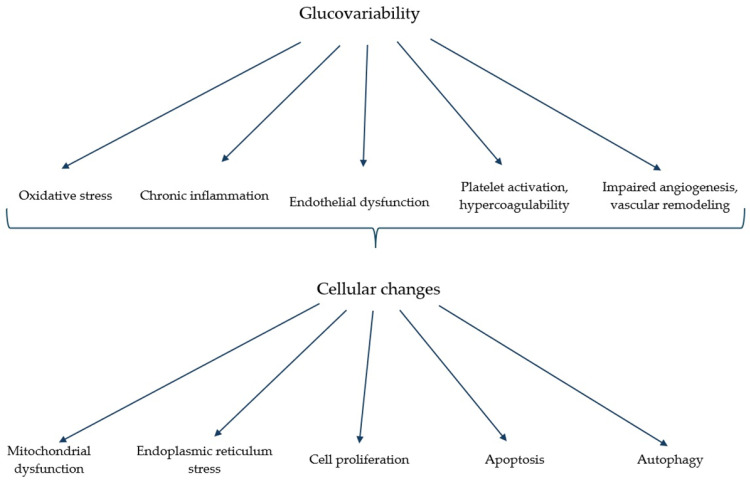
Glucovariability biochemical, pathophysiological, and cellular changes.

The detrimental effects of hypoglycemia manifest in decreased nitric oxide (NO) levels, increased oxidative stress, the production of free oxygen radicals, endothelial dysfunction, the activation of the sympathoadrenal system, and the stimulation of proinflammatory and procoagulant pathways, all of which enhance the susceptibility to thrombosis, atherosclerosis, and cardiovascular events [16]. Conversely, hyperglycemia leads to glucotoxicity, characterized by oxidative stress, AGE production, activation of alternative metabolic pathways, inflammation, endothelial dysfunction, and ischaemia, resulting in both micro- and macrovascular complications (Figure 1). Several studies showed that in case of higher glycemic variability, the harmful effects of hypo- and hyperglycemia add up, exacerbating vascular damage (Figure 3) [17]. Although patients with T1DM are less frequently affected by obesity, dyslipidemia, or any of the further components of the metabolic syndrome in T2DM, they are also at higher cardiovascular risk compared to the healthy population [7]. In our study, CGM users exhibited significantly lower fasting TG and higher HDL levels, correlating with a reduced cardiovascular risk profile. Our findings suggest that CGM usage in T1DM patients may contribute to a decreased risk of cardiovascular events by improving both glycemic stability and lipid parameters.

The fluctuation in glucose levels and their associated harmful outcomes contribute to neuronal injury. In our study, all cardiovascular autonomic reflex tests showed better values while there was a significant difference between CGM users and non-users regarding heart rate responses to the Valsalva maneuver, with non-CGM users exhibiting fewer physiological parameters. Supporting this, Jun J.E. et al. reported that in T1DM patients using CGM, glucose variability was strongly associated with CAN, independent of mean glucose levels [18]. Nyiraty Sz et al. have also proven that several parameters of glucose variability are abnormal in the presence of CAN [19]. Cardiac autonomic dysfunction is known to increase mortality [20] primarily through impaired cardiac adaptation and the relatively augmented sympathetic tone, resulting in sudden arrhythmia, silent cardiac ischemia, and orthostatic hypotension. Our findings suggest that effective therapy for preventing CAN should not only target average glucose levels but also prioritize glucose stability. Our study design does not allow for detailed differentiation of the underlying pathogenic mechanisms linking improved cardiovascular function to CGM use. Regardless of that, the fact that the more physiologic response is proven by the Valsalva maneuver in patients with CGM highlights the importance of the glucose-measuring method in the prevention of the undesirable consequences of cardiovascular dysfunctions.

Consistent findings were observed during the assessment of the peripheral sensory function of our patients. The calibrated tuning fork test indicated a better vibratory sensory function on the upper and lower extremities in patients with CGM. Similarly, Neurometer measurements revealed lower perception thresholds within the normal range at 2000 Hz stimulating frequency on the median nerve in CGM users, suggesting a better sensory condition in these subjects. Both methods assess the functionality of the large sensory nerve fibers, which were found to be in a more physiologic state in CGM users. Preserving intact vibratory function is critical in preventing vascular complications as impaired vibration sensation, identified at the start of the EURODIAB IDDM Prospective Trial, was shown to be a significant predictor of severe lower extremity complications and mortality [21]. Patients with abnormal vibration thresholds were more likely to develop lower extremity complications, such as ulcers and gangrene, and more frequently required lower extremity bypass surgery or angioplasty. Additionally, these patients faced a six times higher risk of amputation compared to those with normal vibration sensation. The presence of cardiovascular disease further doubled the risk of large-fiber damage [22]. Our findings highlight the pathophysiological significance of improved glycemic and lipid control facilitated by CGM. The use of CGM appears to play a crucial role in supporting better peripheral sensory function, emphasizing its importance in the comprehensive management of T1DM. As the broader application of digital health technology is currently more and more frequently recommended [23], its beneficial effect on the outcomes of the whole T1DM population is estimated.

Our study had some limitations. One notable limitation was the significant age difference between the two groups. This disparity might be attributed to younger patients being more adept at using smart devices and demonstrating more openness to apply advanced diagnostic and therapeutic technologies. It should be noted, however, that there was no significant difference in the duration of diabetes, which is one of the most important risk factors for the development of neuropathy in T1DM. Also, all our patients were adherent and followed the carbohydrate restriction in their diet, as calculated individually. Another limitation is the cross-sectional design of our study. In addition, this study is based on laboratory parameters, CGM data, and functional neuronal tests, without taking into account participants’ detailed lifestyle characteristics or medications for diabetes or other comorbid conditions. While the beneficial effect of CGM on glycemic control was evident in improved HbA_1c_ levels, other potential outcomes or confounding variables were not assessed, limiting the scope of our findings.

## 4. Materials and Methods

### 4.1. Patients

Patients were recruited in a diabetology outpatient clinic (Department of Medicine, Albert Szent-Györgyi Medical School, University of Szeged, Szeged, Hungary). The inclusion criteria were a diagnosis of T1DM and an age between 18–65 years. During the visits, blood sampling, neuropathy tests, and the collection of CGM data were performed. Patients were divided into two groups based on whether they used CGM or not.

A total of 61 patients (age: 42.5 ± 1.8 years, DM duration: 22.8 ± 1.6 years, BMI: 25.3 ± 0.9, HbA_1c_: 8.1 ± 0.2%; mean ± SE) were studied (Table 1). In total, 24 patients used CGM sensors regularly (age: 35.7 ± 2.5, DM duration: 20.0 ± 1.8, BMI: 24.5 ± 0.8, HbA_1c_: 7.5 ± 0.8%) and 37 did not (age: 45.9 ± 1.8, DM duration: 25.0 ± 1.9, BMI: 26.5 ± 0.8, HbA_1c_: 8.3 ± 0.3%, Table 2).

### 4.2. Evaluating Sensory Dysfunction

Peripheral sensory nerve function was assessed by a Neurometer (MSB Ltd., Balatonfüred, Hungary) and a calibrated tuning fork was applied to the upper and lower extremities. The Neurometer device was designed to quantify the sensory function of different types of nerve fibers and provides a simple, non-invasive measurement of peripheral sensory function [24]. A transcutaneous low voltage electric sine wave stimulation was delivered on the upper and lower limbs and the current perception threshold (CPT) values were determined. In this study, the sensory functions of the median and peroneal nerves on the left side were tested. The surface electrodes were fixed on the terminal phalanx of the index and the great toes. The electrodes were positioned only on intact skin. The amplitude of the delivered stimuli ranged from 0.01 to 9.99 mA. The stimulus was gradually increased until a sensation was reported by the patient; then, short stimuli (2 to 5 s) were applied at progressively lower amplitudes until a consistently minimal threshold for detection was found. The CPT values of the upper and lower limbs were detected at three different stimulating frequencies (2 kHz, 250 Hz, and 5 Hz) to ensure the separate testing of the large and small sensory fibers.

The 128 Hz Rydel–Seiffer graduated tuning fork was used to evaluate the vibration sense at the ulnar styloid process and at the interphalangeal joint of the hallux of the right and left legs [25]. The normal range was declared as 7 to 8, 6 was classified as borderline, and scores between 1 and 5 indicated an impaired sense of vibration.

### 4.3. Assessment of Autonomic Neuropathy

Autonomic function was characterized by Ewing’s standard cardiovascular reflex tests in this study [26]. The Ewing tests are the gold standards for diagnosing autonomic dysfunction; they provide non-invasive, clinically relevant, standardized, and reproducible data on autonomic functions. Reflex tests were performed by measuring the blood pressure and obtaining continuous six-lead ECG signals. The signals were digitized with a multichannel data acquisition system (CA-12 v.2.85 software, 2021, MSB Ltd., Balatonfüred, Hungary). Parasympathetic dysfunction was examined by measuring deep breathing tests, the Valsalva ratio (VR), and the 30:15 ratio while sympathetic nervous system impairment was tested by assessing orthostatic blood pressure drop on standing up. As the handgrip test is becoming a marker of hypertension and its complications more and more, we did not use it in our evaluation [27].

### 4.4. Laboratory Measurements

The metabolic state was evaluated by measuring TG, HDL, LDL and total cholesterol, HbA_1c_ levels. The reference ranges for these parameters in our laboratory are: TG: <1.70 mmol/L, HDL: >1.40, LDL <3.0, and total cholesterol: <5.20.

### 4.5. Statistical Analysis

During data analysis, continuous variables were expressed as means and standard error (mean ± SE) while categorical variables were expressed as frequencies and percentages (*n*, %). Univariate analyses were performed using independent sample *t*-tests and Pearson correlation coefficients (r). Statistical analyses were performed using PAST 4.09 (University of Oslo, Oslo, Norway). During the analyses, *p* values < 0.05 were considered statistically significant.

### 4.6. Ethics

This study was performed in line with the Good Clinical Practice guidelines and the Declaration of Helsinki in its latest form. The study protocol was approved by the Regional and Institutional Review Board of Human Investigations at the University of Szeged (67/2022-SZTE, approval date: 7 June 2023). All subjects signed an informed consent form for this study.

## 5. Conclusions

In summary, our findings suggest that better glycaemic control, as reflected by HbA_1c_ levels, is associated with lower TG levels in T1DM patients. Those using CGM experienced further benefits, including more favorable TG and HDL levels. In addition, these patients had significantly better cardiovascular autonomic and peripheral sensory function. The advantage of CGM use might be realized through the better glycemic control and the lower lipid levels as well. The combined metabolic effects underline the potential of CGM as a valuable tool not only for managing diabetes but also, as a novel finding, for preventing or postponing the development of neural dysfunction as well. This highlights the promise of CGM technology to contribute to long-term cardiovascular and neurological health in T1DM patients.

## Figures and Tables

**Table 1:** Patients’s data.

T1DM Patients	
Number of patients	61
Age (years)	42.5 ± 1.8
Duration of diabetes (years)	22.8 ± 1.6
BMI (kg/m^2^)	25.2 ± 0.9
HbA_1c_ (%)	8.1 ± 0.2

**Table 2:** Data on CGM users and non-users.

Patients	CGM Users	CGM Non-Users	*p*-Value
Number of patients	24	37	/
Age (years)	35.7 ± 2.5	45.9 ± 1.8 *	0.01
Duration of diabetes	20.0 ± 1.8	25.0 ± 1.9	0.1
BMI (kg/m^2^)	24.5 ± 0.8	26.5 ± 0.8	0.1
HbA_1c_ (%)	7.5 ± 0.8	8.3 ± 0.3 *	0.04

*: *p* < 0.05.

**Table 3:** Results of the cardiovascular autonomic function tests in CGM users and non-users.

Patients	CGM Users	CGM Non-Users	*p*-Value
Heart rate response to deep breathing (beats/min)	24.7 ± 2.9	18.9 ± 1.9	0.22
Valsalva ratio	1.4 ± 0.06	1.2 ± 0.04 *	0.045
30:15 ratio	1.1 ± 0.03	1.1 ± 0.01	0.32
Orthostasis (mmHg)	4.7 ± 1.35	7.7 ± 1.9	0.26

*: *p* < 0.05.

**Table 4:** Peripheral sensory function in CGM users and non-users by the Neurometer assessing the threshold of the current sensations at the median and peroneal nerves at three different stimulating frequencies (2 kHz, 250 Hz, 5 Hz).

Neurometer	CGM Users	CGM Non-Users	*p*-Value
n.medianus 2000 Hz	224 ± 21.2	290 ± 17.7 *	0.01
n.medianus 250 Hz	87.2 ± 11.8	116 ± 11.4	0.08
n.medianus 5 Hz	59.1 ± 7.8	63.6 ± 7.5	0.38
n.peroneus 2000 Hz	399.1 ± 33.8	407.3 ± 20.1	0.34
n.peroneus 250 Hz	189.2 ± 21.7	200.7 ± 12.6	0.42
n.peroneus 5 Hz	127.8 ± 17.4	123.2 ± 9.3	0.46

*: *p* < 0.05.

**Table 5:** Vibratory perception threshold evaluated by calibrated tuning fork in CGM users and non-users.

Calibrated Tuning Fork	CGM Users	CGM Non-Users	*p*-Value
right radius	7.4 ± 0.1	7.1 ± 0.1 *	0.01
left radius	7.0 ± 0.4	6.9 ± 0.2	0.35
right hallux	7.2 ± 0.2	6.1 ± 0.3 *	0.005
left hallux	6.7 ± 0.4	6.1 ± 0.3	0.32

*: *p* < 0.05.

**Table 6:** Metabolic parameters in CGM users and non-users.

Metabolic Parameters	CGM Users	CGM Non-Users	*p*-Value
HbA_1c_ (%)	7.5 ± 0.8	8.3 ± 0.3 *	0.04
Trigliceride (mmol/L)	0.9 ± 0.1	1.2 ± 0.1 *	0.034
Cholesterol (mmol/L)	4.7 ± 0.2	4.9 ± 0.1	0.13
LDL (mmol/L)	2.6 ± 0.1	2.9 ± 0.1	0.07
HDL (mmol/L)	1.7 ± 0.07	1.4 ± 0.06 *	0.02

*: *p* < 0.05.

## Data Availability

Our figures contain mean ± SE data of our T1DM patients.

## Abbreviations

The following abbreviations are used in this manuscript:

AGE	Advanced glycated end products
BMI	Body mass index
CAN	Cardiovascular autonomic neuropathy
CGM	Continuous glucose monitoring
COX-2	Cyclooxygenase-2
CPT	Current perception threshold
DSPN	Distal symmetric polyneuropathy
HbA_1c_	Glycated hemoglobin
HDL	High-density lipoprotein
IL-1	Interleukin-1
IL-6	Interleukin-6
LDL	Low-density lipoprotein
LPL	Lipoprotein lipase
PAI-1	Plasminogen activator inhibitor-1
TAR	Time above range
TBR	Time below range
TIR	Time in range
T1DM	Type 1 diabetes mellitus
T2DM	Type 2 diabetes mellitus
TG	Triglyceride
TNF-a	Tumor necrosis factor-alpha
TXA	Thromboxan A
TXAB-2	Thromboxan B-2
VLDL	Very low-density lipoprotein
VR	Valsalva ratio
